# Headache in the first manifestation of Multiple Sclerosis – Prospective, multicenter study

**DOI:** 10.1002/brb3.852

**Published:** 2017-11-16

**Authors:** Marcel Gebhardt, Peter Kropp, Tim P. Jürgens, Frank Hoffmann, Uwe K. Zettl

**Affiliations:** ^1^ Krankenhaus Martha‐Maria Halle‐Dölau Halle Germany; ^2^ Institute of Medical Psychology and Medical Sociology Medical Faculty University of Rostock Rostock Germany; ^3^ Department of Neurology University Medical Center Rostock Rostock Germany; ^4^ Department of Neurology Neuroimmunological Section University of Rostock Rostock Germany

**Keywords:** clinically isolated syndrome, headache, migraine, multiple sclerosis, radiologically isolated syndrome

## Abstract

**Objectives:**

Multiple sclerosis (MS) is the most frequent immune‐mediated inflammation of the central nervous system that can lead to early disability. Headaches have not been considered as MS‐related symptoms initially, whereas higher prevalence rates were reported since 2000. Postmortem histological analyses of MS patients’ brains revealed lymphoid follicle‐like structures in the cerebral meninges which suggest a possible pathophysiological explanation for the high headache prevalence in MS. The aim of this study was to evaluate headache characteristics during the first clinical event of MS.

**Methods:**

In a prospective, multicenter study, 50 patients with the diagnosis of CIS or MS were recruited. All participants were screened for the presence of headache within the last 4 weeks with help of the Rostock Headache Questionnaire (Rokoko).

**Results:**

Thirty‐nine of fifty questioned patients (78%) reported headaches within the last 4 weeks. Most patients suffered from throbbing and pulsating headaches (25, 50%), 15 (30%) reported stabbing, 14 (28%) dull and constrictive headaches.

**Conclusions:**

Headaches were prevalent in 78% of patients in our population with newly diagnosed CIS and MS. It is among the highest prevalence rates reported so far in patients with CIS or MS. Thus, headache, especially of a migraneous subtype, is a frequent symptom within the scope of the first manifestation of multiple sclerosis. If it were possible to define a MS‐typical headache, patients with these headaches and with typical MRI results would be classified as CIS or early MS instead of radiologically isolated syndrome and treated accordingly with an immunomodulatory therapy.

## 1 INTRODUCTION

Multiple sclerosis (MS) is the most frequent immune‐mediated inflammation of the central nervous system (CNS) that can lead to early disability (Flachenecker et al., 2008). In 2013, the worldwide MS prevalence was 2.3 million (Browne et al., 2014). The mean age at the first clinical manifestation of MS typically lies between the 20th and 40th year. However, there are also known cases with occurrence from infancy up to well after the 80th year. As in migraine, females are affected 2–3 times more often than males (Flachenecker et al., 2005).

While many symptoms such as paresis, paresthesias and numbness, ataxia, visual disturbances, neuro‐urological dysfunction, neuropsychological deficits, and fatigue often appear in the course of MS, headaches are not regarded as typical early MS symptoms or MS‐related symptoms (Möhrke, Kropp, & Zettl, 2013; Zettl, Stüve, & Patejdl, 2012). Till the 1990s relatively low prevalence rates for headaches in the course of MS were found ranging between 4% and 37.5% (Abb & Schaltenbrand, 1956; Bonduelle & Albaranes, 1962; Clifford & Trotter, 1984; Freedman & Gray, 1989; Poser, Presthus, & Horsda, 1966; Watkins & Espir, 1969), whereas higher prevalence rates of up to 64% were reported since 2000 (Boneschi et al., 2008; D'Amico et al., 2004; Kister et al., 2010; Möhrke et al., 2013; Nicoletti et al., 2008; Putzki et al., 2009; Vacca et al., 2007; Villani et al., 2008).

This high prevalence of headache raises the question whether it is really a comorbidity of two independent diseases or whether the headache could be a primary symptom of MS. Clinically and therapeutically this would be highly relevant, as the presence of headache alone would allow the diagnostic classification as clinically isolated syndrome (CIS) or early MS (Polman et al., 2011) instead of radiologically isolated syndrome (RIS) and could be helpful to assess disease activity (Lublin, 2014). RIS is defined by typical MS lesions found in magnetic resonance imaging (MRI), without the presence of clinical MS symptoms (Okuda et al., 2009).

Postmortem histological analyses of the brains in MS patients revealed the presence of lymphoid follicle‐like structures in the cerebral meninges resulting in meningeal inflammation as a possible pathophysiological explanation for the high prevalence of headache, especially migraine (Magliozzi et al., 2007). It is striking that these tertiary, in particular, B‐cell follicles were found in 41.4% of the patients with secondary progressive MS (SPMS) (Howell et al., 2011; Magliozzi et al., 2010). In addition, there is also a higher number of macrophages, T‐ and B cells in patients with SPMS. SPMS is marked by transition into a continuously progressive form of the disease without the initial relapsing‐remitting phenotype anymore associated with an increase in degeneration. An even higher degree of meningeal inflammation (and consecutively headache) is to be expected with patients in the early stage of the relapsing‐remitting MS (RRMS) or CIS as inflammation is typically predominant here. However, due to the low impairment and mortality in the early stage, postmortem analyses for these patients have not been performed.

Möhrke et al. (2013) reported that MS patients with headaches are younger, more likely female, and less severely affected in their motor functions than those without. The patient cohort consisted of patients with relapsing‐remitting as well as progressive course, but only three patients with CIS.

The aim of this study was to evaluate headache characteristics during the first clinical event when the initial diagnosis of a CIS or MS was made.

## 2 METHODS

In a prospective, multicenter study 50 consecutive patients with the initial diagnosis of CIS or MS and an occurrence of neurological symptoms within the last 6 months were recruited in the Department of Neurology of Martha‐Maria Hospital and in four private neurological practices in Halle, Germany. The current diagnostic criteria by McDonald (Polman et al., 2011) were met based on an extensive history, clinical examination, MRI of the CNS, and cerebrospinal fluid (CSF) at the time of the first clinical manifestation of neurological symptoms. Exclusion criteria were concomitant diseases known to cause secondary headaches (such as cerebral hemorrhage). There were no screening failures or drop outs.

All study participants underwent in the course of the diagnostic process a detailed and structured clinical interview about their case history and they were asked to complete a semistructured interview covering headache frequency, duration, character, localization, and the presence of accompanying symptoms within the last 4 weeks before hospitalization with help of the Rostock Headache Questionnaire (Rokoko), a validated tool to screen primary headaches (Müller et al., 2014). It allows classification of headaches into migraine with and without aura, tension‐type headache (TTH) and cluster headache. Concerning optic neuritis, we asked especially for pain localized in retro‐orbital, orbital or frontal region, or aggravation by eye movement, so we separated the symptoms from other forms of headache.

**Figure 1:**
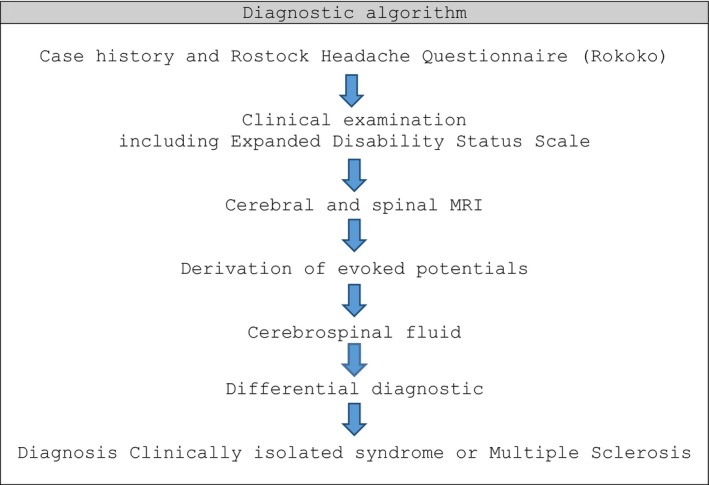
Diagnostic algorithm

Afterward the participants underwent a clinical‐neurological investigation, including the evaluation of disease severity on the Expanded Disability Status Scale (EDSS) (Kurtzke, 1983). Then, a MRI of the central nervous system and a CSF analysis were done. In addition, visual and somatosensory‐evoked potentials were recorded as well as transcranial magnetic stimulation and we determined relevant antibodies for differential diagnostic (Figure 1).

This study was approved by the ethics committee of the medical association Saxony‐Anhalt (no. 7/15).

## 3 RESULTS

Thirty‐nine women (78%) and 11 men (22%) were included. The mean age of the patients was 32.0 years (SD 9.4, min 18, max 54). The latency between the first onset of clinical symptoms to study inclusion amounted from 5 days to a maximum of 6 months. The mean EDSS value was to 1.9 (SD 1.6) (Table 1).

**Table 1:** Sociodemographic and neurological parameters

	*N* a	[%]	Mean	SD
Patients	50			
Gender				
Male	11 (4)	22		
Female	39 (8)	78		
Age			32.0	9.4
EDSS			1.9	1.6
Symptomatic				
Monosymptomatic	24	48		
Polysymptomatic	26	52		
Symptoms^b^				
Headache	39	78		
Paresthesia and numbness	30	60		
Paresis	16	32		
Optic neuritis	14	28		
Brainstem, cerebellar symptoms	9	18		
Neuro‐urological dysfunction	2	4		
Dysarthria	2	4		

a Positive family history for headaches.

b Multiple appointment possible.

Thirty‐nine of fifty patients (78%) reported headaches within the last 4 weeks. According to the criteria of the RoKoKo, seven patients (14%) suffered from TTH, five (10%) from migraine (three without aura, two with aura), 18 (36%) from migraine‐like headaches, and nine (18%) from unclassifiable headaches.

Twenty‐three patients (46%) reported recurrent headaches only, seven (14%) of permanent, and eight (16%) of both recurrent and permanent headaches. Most patients suffered from throbbing and pulsating headaches (25, 50%), 15 (30%) reported stabbing, and 14 (28%) dull and constrictive headaches (Table 2).

**Table 2:** Headache phenomenology

	*N*	[%]
Pain frequency		
Recurrent	23	46
Permanent	7	14
Recurrent and permanent	8	16
Other than that	1	2
Pain character^a^		
Throbbing and pulsating	25	50
Stabbing	15	30
Dull and constrictive	14	28
Burning	3	6
Nagging	5	10
Duration		
<1 hr	2	4
1–3 hr	13	26
4–72 hr	28	56
>72 hr	3	6
Headache medication		
Never	21	42
1–4×/month	15	30
5–9×/month	5	10
>=10×/month	9	18
4 weeks after glucocorticosteroid therapy		
Small improvement	11	28.2
Substantial improvement	10	25.6
Completely remitted	8	20.5
Change in location and character	1	2.6
No change	9	23.1

a Multiple appointment possible.

Headaches lasted between 4 and 72 hr in 28 patients (56%). Two patients (4%) also reported having <1 hr of headaches, 13 patients (26%) between 1 and 3 hr and three patients (6%) longer than 72 hr. Eight patients (16%) even reported of longer than 14 days lasting headaches during the last 4 weeks.

Twenty‐one of fifty patients (42%) had taken no headache‐specific medication during the preceding 4 weeks. Fifteen patients (30%) had taken medication for 1–4 days, five (10%) for 5–9 days, and nine patients (18%) for 10 and more days during the last 4 weeks.

A total of 12 patients (24%) reported a positive family history for headaches.

Headaches were the most frequent neurological symptom at the time of the clinical manifestation of the inflammatory CNS disease (CIS, RRMS) (78% of patients).

Thirty patients (60%) suffered from paresthesia (“tingling”) and numbness, 16 (32%) had central paresis. In 14 patients (28%) the disease began with an optic neuritis, in nine (18%) with brainstem or cerebellar symptoms, in two patients (4%) with neuro‐urological dysfunction, and in two patients (4%) with a dysarthria.

All patients received a high‐dose intravenous glucocorticosteroid (GCS) treatment for 5 days with 1000 mg methylprednisolone per day. Four weeks after GCS therapy 11 of the 39 headache patients (28.2%) reported a small improvement of headaches, 10 (25.6%) a substantial improvement, and in eight (20.5%) patients that the headaches remitted completely. Nine patients (23. 1%) reported no change after the GCS therapy and one patient (2. 6%) noted only a change in headache location and character.

## 4 DISCUSSION

Headache prevalence was as high as 78% in our group of patients and is therefore among the highest found in patients with CIS or MS. While in other studies, MS patients were examined regardless of their disease duration and course (Abb & Schaltenbrand, 1956; Bonduelle & Albaranes, 1962; Boneschi et al., 2008; Clifford & Trotter, 1984; D'Amico et al., 2004; Freedman & Gray, 1989; Kister et al., 2010; Möhrke et al., 2013; Nicoletti et al., 2008; Poser et al., 1966; Putzki et al., 2009; Vacca et al., 2007; Villani et al., 2008; Watkins & Espir, 1969), well‐characterized patients in the early stages of the disease (namely within the first 6 months after the first manifestation of clinical symptoms) were included in this study. In other studies the mean duration of MS ranged between 14 years (Vacca et al., 2007), respectively, 13.4 years (D'Amico et al., 2004) and 8.8 years (Villani et al., 2008). Compared to the already high 1‐year headache prevalence of 62.5% in the general German population (Straube et al., 2013), it was even higher in our selected population. In addition, it was the most frequent neurological symptom reported by patients, followed by paresis, paresthesia and hypesthesia, optic neuritis, and brainstem or cerebellar symptoms. These further symptoms were also found in other studies in the first manifestations of the disease (Poser, Raun, & Poser, 1982; Weinshenker et al., 1989).

The observed high prevalence of headaches, especially of a migraineous subtype, could be due to a shared pathophysiology. Migraine, which affects significantly more females than males (like MS), has been associated with meningeal inflammation resulting in nociceptive trigeminal activation (Levy, 2009). It has been suggested that inflammatory activity is highest early in the course of MS which would well explain a large overlap with especially migraineous headaches. It is tempting to speculate that headache could in fact be an early symptom of MS itself and not merely coincidental.

Interestingly, Granberg et al. found that in half of the patients with RIS, headaches were the reason for the MRI investigation leading to the incidental diagnosis of RIS (Granberg, Martola, Kristoffersen‐Wiberg, Aspelin, & Fredrikson, 2013). In two of three cases, a progression of MRI lesions can be observed during the next 2 to 5 years, while in the same period, one of three cases fulfill the criteria for “classical” CIS or MS (Granberg et al., 2013). Of great importance is the exact classification between RIS and CIS/RRMS, because studies show that the magnitude of the T2 lesions and signs of neurodegeneration do not differ from RIS patients to patients with early RRMS (Azevedo et al., 2015; De Stefano et al., 2011). These data suggest that if it were possible to define a MS‐typical headache, with the aid of other examinations, patients with these headaches and with typical MRI results would be classified as CIS or early MS instead of RIS and treated with an immunomodulatory therapy (Comi et al., 2009; Kappos et al., 2006; Miller & Leary, 2009).

More than 50% of our CIS patients suffer from headaches which do not fulfill the formal diagnostic criteria of primary headaches. Most often, recurrent pain of pulsating‐throbbing character was reported lasting between 4 and 72 hr. It could be classified as probable migraine (36%) according to the ICHD‐III (Headache Classification Committee of the International Headache Society, 2013), because they fulfill three of four diagnostic criteria required for a formal diagnosis of migraine and at the same time do not fulfill the criteria of another primary headache. Five patients (10%) even fulfilled all diagnostic criteria of a migraine, so that almost half of all patients suffer from a migraine or a probable migraine; however, only seven (14%) suffer from TTH (test sensitivity/specificity of the RoKoKo of 0.87/0.51 for migraine without aura, 0.71/0.95 for migraine with aura and 0.57/0.93 for TTH). These results are comparable with other studies. In a work of Tabby, Majeed, Youngman, & Wilcox (2013), throbbing headaches were found in 52.9% patients as the most frequent form in 72 MS patients with headaches. D'Amico et al. (2004) found a substantial number of MS patients reported headaches that were classified as probable migraine, because they did not fulfill all diagnostic criteria of a migraine. These also occurred more frequently in patients with RRMS, while TTH was associated with the progressive course of the disease. The strongly differing findings of previous studies on migraine prevalence in MS patients between 19.8% and 82% (Nicoletti et al., 2008; Vacca et al., 2007) and TTH prevalence between 12.2% and 55.2% (D'Amico et al., 2004; Villani et al., 2008) appear to correlate with MS duration.

Pakpoor, Handel, Giovannoni, Dobson, & Ramagopalan (2012) found within the scope of a meta‐analysis that MS patients suffer from migraine more than twice as often as healthy controls. Other studies support a close co‐occurrence of both diseases: in a study of Kister, Munger, Herbert, & Ascherio (2012) patients with migraine had a 39‐fold higher risk of developing MS, while Kruit, van Bucehm, Launer, Terwindt, & Ferrari (2010) reported a correlation of migraine attack frequency with the number of white‐matter lesions. Moreover, it appeared that MS patients with migraine suffered from more relapses than MS patients without migraine (Elliot, 2007; Kister et al., 2010). Another striking similarity between MS and migraine is the high prevalence in young women and the protective effect of pregnancy and lactation. Also undulating courses, episodes with high disease activity over many years and intermittent phases of high disease activity are often observed in both disorders. This raises the question whether migraine headache observed in a large group of MS patients represents a symptomatic headache mimicking migraine or represents an activation of genuine migraine pathology with inflammatory changes as the trigger.

Interestingly, 29 of 39 patients (74.4%) noted an improvement of the headaches following steroid therapy after 4 weeks. Although some studies have questioned the efficacy of steroids in migraine (Fiesseler et al., 2011), there is substantial evidence that steroids interfere with CGRP release (Neeb, Hellen, Hoffmann, Dirnagl, & Reuter, 2016; Neeb et al., 2015). In status migrainosus, high dose of GCS were effective additively (Rozen, 2002). Therefore, it would be interesting to examine whether patients experience a long‐term improvement of their headaches after short‐time therapy with GCS.

From a pathophysiological perspective, the meningeal inflammation described by Maggliozi et al. involving B‐cell and T‐cell activation in MS patients could also be the cause of headache manifestation. This would also explain why the prevalence of headaches is so high at the beginning of the disease.

Despite the intriguing findings, our study has some shortcomings. Headache characteristics were assessed with the Rokoko‐questionnaire only, which has been shown to have a high diagnostic accuracy (Kurtzke, 1983). However, a relatively large group of patients with headaches could not be allocated to either migraine or tension‐type headache. In a structured clinical interview carried out by a headache expert this could have had reached a higher sensitivity and specificity. In addition, a previous personal history of headaches was not assessed, so that we could not ascertain whether the reported headaches developed de novo in temporal relation to the neurological symptoms leading to the diagnosis of CIS or MS or whether they were just an exacerbation or reoccurrence of a preexisting primary headache. Also the emotional stress prior to the hospitalization could be a reason for exacerbation of migraine and so a potential bias.Due to the lack of an appropriate control group it is difficult to assess whether prevalence rates in our sample are indeed higher than in the general population. However, setting up a matched control group is problematic and would require a large prospective epidemiological study.

## 5 CONCLUSION

Headaches were prevalent in 78% of patients in our population with newly diagnosed CIS and MS. It is among the highest prevalence rates reported so far in patients with CIS or MS. Thus, headache, especially of a migraneous subtype, is a frequent symptom within the scope of the first manifestation of multiple sclerosis. However, it is not regarded as MS‐related or typical symptom. If it were possible to define a MS‐typical headache, patients with these headaches and with MS‐typical MRI results would be classified as CIS or early MS instead of RIS and treated accordingly with an immunomodulatory therapy. This kind of MS‐typical headache seems to be very similar to migraine, because we often found recurrent pain of pulsating‐throbbing character, lasting between 4 and 72 hr, that does not fulfill the criteria of migraine. Separating these cases from “classical” migraine may help the results of studies, which could show strong differences between MS and migraine in MR morphology (Absinta et al., 2012). According to this it could be an important aim of future MR‐studies to work out differences in MR findings between MS patients with and without headache.

## CONSENT FOR PUBLICATION

Not applicable.

## AVAILABILITY OF DATA AND MATERIAL

The datasets used and analyzed during this study are available from the corresponding author on reasonable request.

## DISCLOSURES

All authors declare that they have no competing interests.

## AUTHORS' CONTRIBUTIONS

Not applicable.
